# Supplementary material for: Feed-forward loops by NR5A2 ensure robust gene activation during pre-implantation development
Source: Development. 2026 Jan 8;153(1):dev205059. doi: 10.1242/dev.205059 (PMC12848575; doi:10.1242/dev.205059)
Supplement: Supplementary information [file develop-153-205059-s1.pdf]

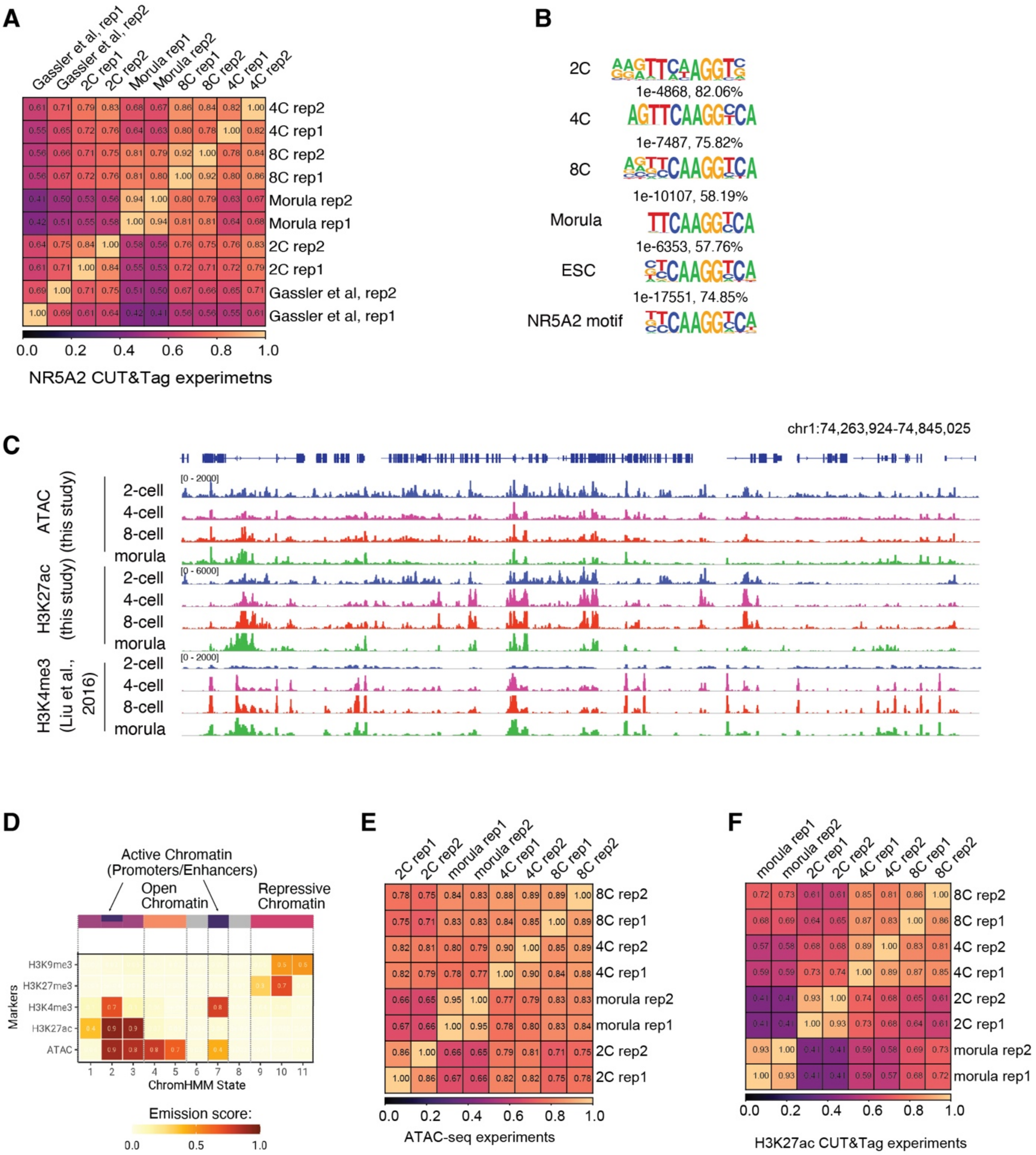

Fig. S1. Mapping of NR5A2 chromatin binding and chromatin states.

(**A**) Correlation matrix (Pearson correlation) of NR5A2 CUT&Tag with a 1 kb window. (**B**) DNA motifs identified by HOMER *de novo* motif analysis of NR5A2 peaks at each developmental stage. The NR5A2 motif (MA0505.1) from the JASPAR database is shown as a reference. P-values and percentage of motif presence in peaks are shown. (**C**) IGV snapshot showing ATAC-seq and H3K27ac CUT&Tag signals across developmental stages: 2-cell (blue), 4-cell (purple), 8-cell (red), and morula (green). Publicly available H3K4me3 ChIP-seq data (Liu et al., 2016) is shown. (**D**) Chromatin state annotation by ChromHMM. (**E, F**) Correlation matrix (Pearson correlation) of ATAC-seq (**E**) and H3K27ac CUT&Tag (**F**) comparing between replicates and cell stages.

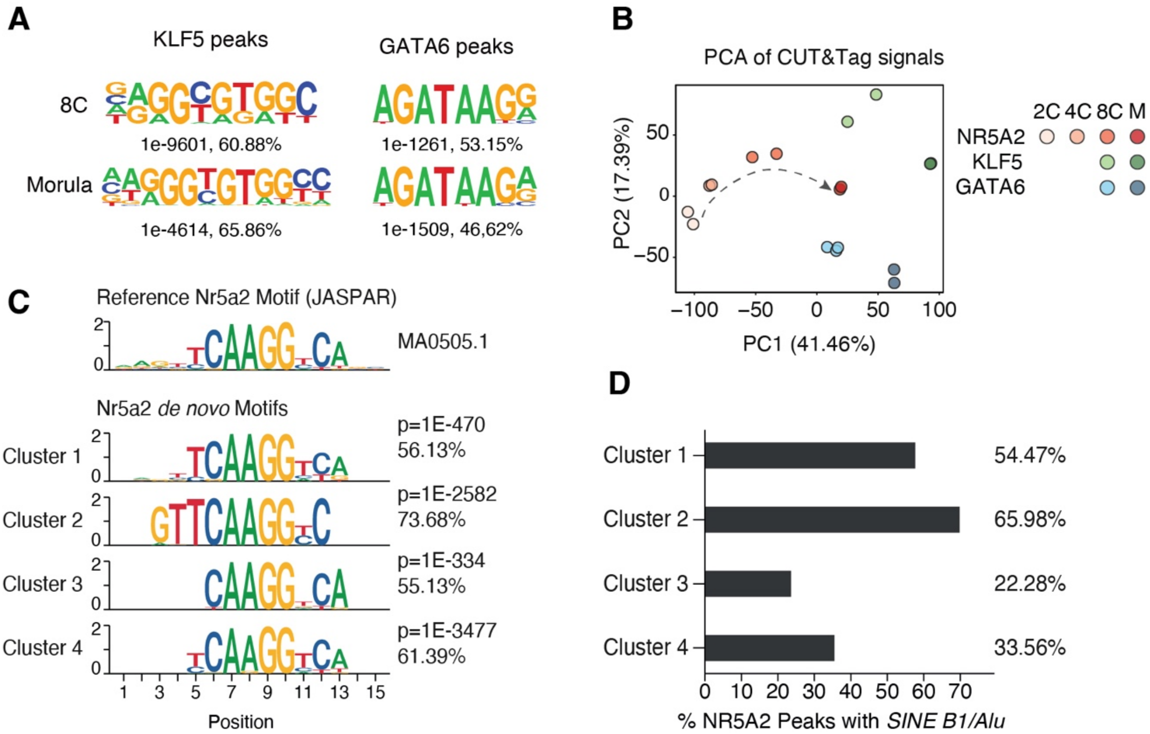

Fig. S2. KLF5 and GATA6 binding at the 8-cell and morula stages.

(**A**) DNA motifs identified by HOMER *de novo* motif analysis of KLF5 and GATA6 peaks at the 8-cell and the morula stages. P-values and percentage of motif presence in peaks are shown. (**B**) Principal component analysis of NR5A2, KLF5, and GATA6 CUT&Tag profiles at each stage. The allow shows the trajectory of NR5A2 binding from the 2-cell to the morula stage. (**C**) *De novo* motifs of NR5A2 peaks in different clusters. P-values and percentage of motif presence in peaks are shown**.** (**D**) Percent of peaks from each cluster of NR5A2 binding sites at morula stage that contain *SINE B1/Alu* within 250 bp from their centers.

Development • Supplementary information

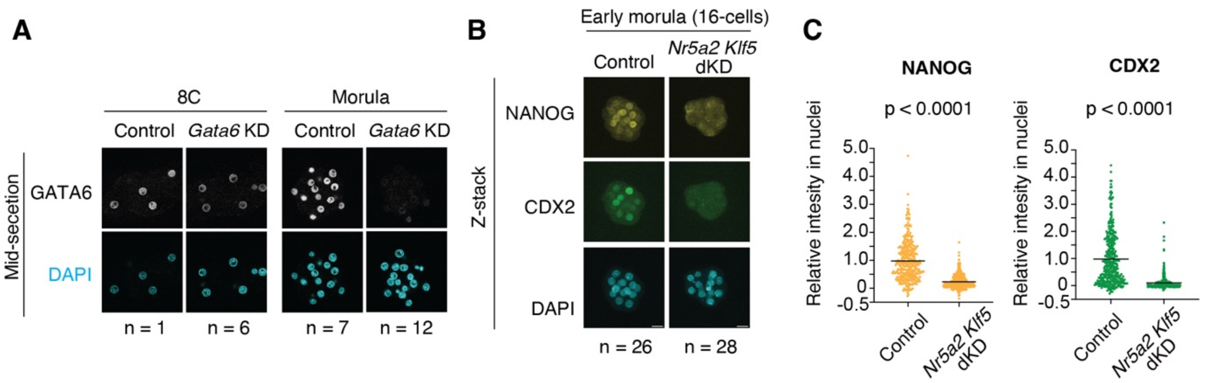

Fig. S3. Immunofluorescence analysis with knockdown embryos.

(**A**) Representative images of immunostaining analysis showing GATA6 (grey) and DAPI (cyan) in 8-cell embryos. Both signals are shown in mid-section images. The number of embryos examined (n) from one experiment is indicated. (**B, C**) Representative images (**B**) and quantification data **(C**) of immunostaining analysis showing NANOG (yellow), CDX2 (green), and DAPI (cyan) in early morula (16-cells) embryos. Scale bars, 20 μm. Both signals are shown in z-stack images. The number of embryos examined (n) from two independent experiments is indicated. Bars overlaid on the plots indicate means. P values (t-test, two-sided) are shown.

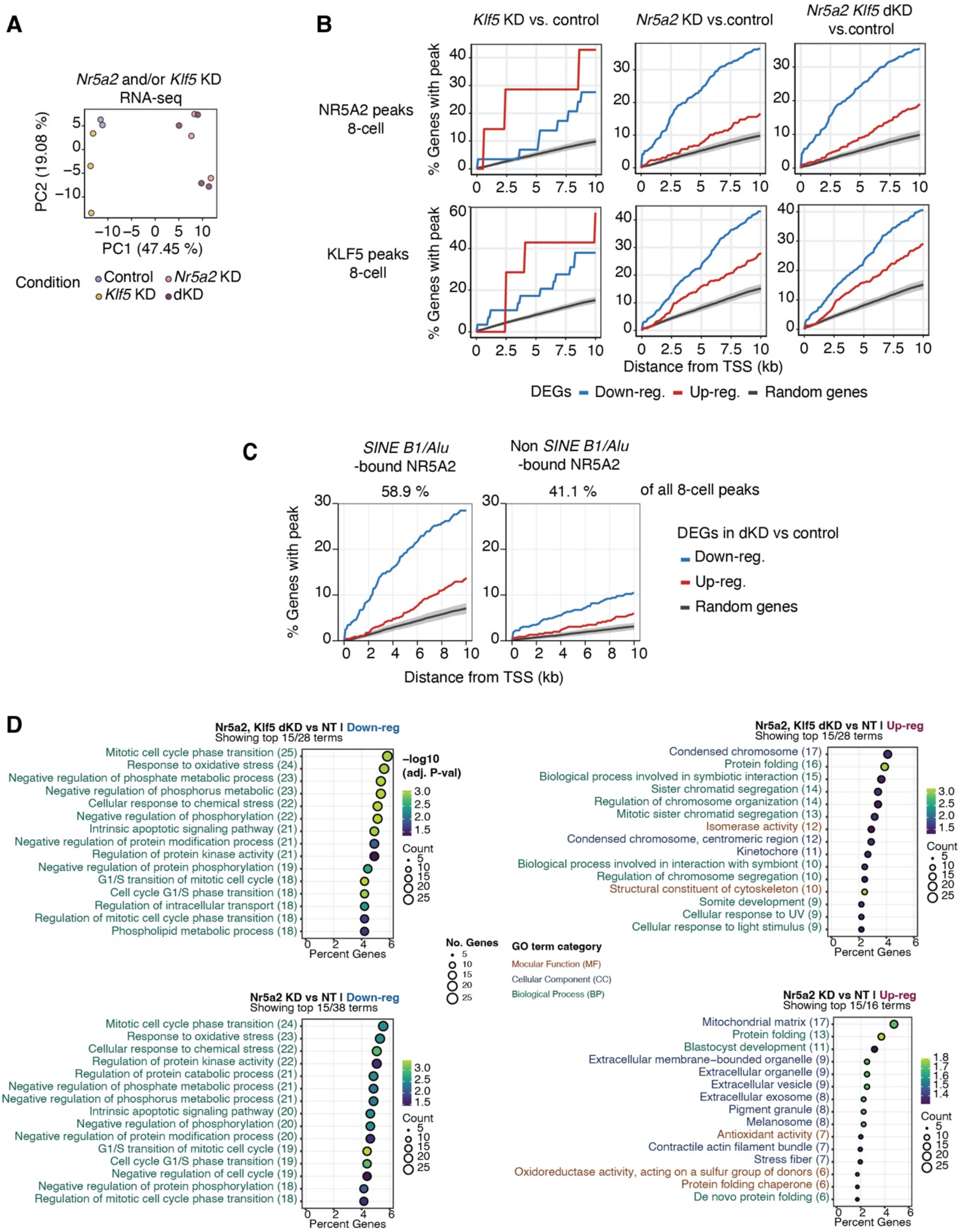

Fig. S4. Transcriptional changes at the 8-cell stage upon siRNA-mediated KD.

(**A**) Principal component analysis of RNA-seq data. Each colored dot indicates a different condition. (**B**, **C**) Cumulative percentage of differential expressed genes (DEGs) containing transcription factor binding sites within certain upstream distances from their transcription start site. (**B**) Percentage of DEGs from different experiment (row) that has NR5A2 and KLF5 binding upstream (column). (**C**) Percentage of DEGs from *Nr5a2 Klf5* dKD condition that contain *SINE B1/Alu*- and non-SINE B1/Alu- bound NR5A2 peaks (**D**) Gene Ontology analysis of down- and up-regulated genes in *Nr5a2 Klf5* dKD and *Nr5a2* KD groups.

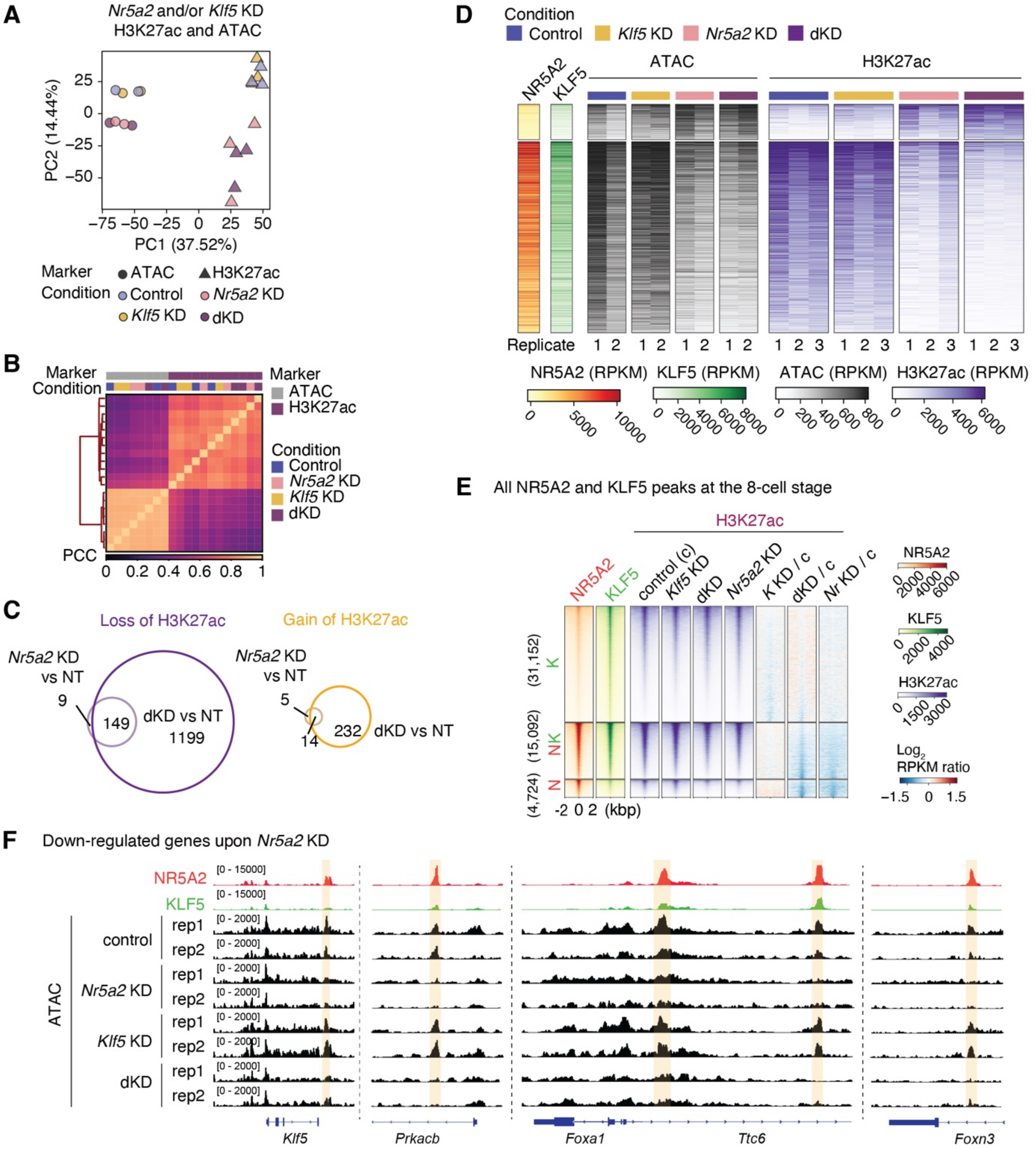

Fig. S5. Chromatin accessibility and H3K27ac profiles at the 8-cell stage upon siRNA-mediated KD.

(**A**) Principal component analysis of H3K27ac CUT&Tag and ATAC-seq data. Each colored dot or triangle indicates a different condition. (**B**) Pearson correlation analysis of ATAC-seq and H3K27ac CUT&Tag in control and each KD condition with replicates. (**C**) Venn diagrams showing overlapped loss and gain H3K27ac regions in *Nr5a2* KD and *Nr5a2 Klf5* dKD. (**D**) Heatmap showing ATAC-seq and H3K27ac CUT&Tag signals in control, *Klf5* KD, *Nr5a2* KD, and *Nr5a2 Klf5* dKD 8-cell embryos at gain and loss regions. NR5A2 and KLF5 CUT&Tag data are shown on the left. Each replicate of ATAC-seq and H3K27ac CUT&Tag experiment is shown in the column. (**E**) Heatmaps showing enrichment of H3K27ac on all peaks of NR5A2, KLF5, co-bound regions. Log_2 RPKM ratios (*Nr5a2* KD/control, dKD/control, and *Klf5* KD/control) are shown, with data from three biological replicates merged. (**D**) IGV snapshot showing chromatin accessibility near down-regulated genes upon *Nr5a2* KD at the 8-cell stage.

Development • Supplementary information

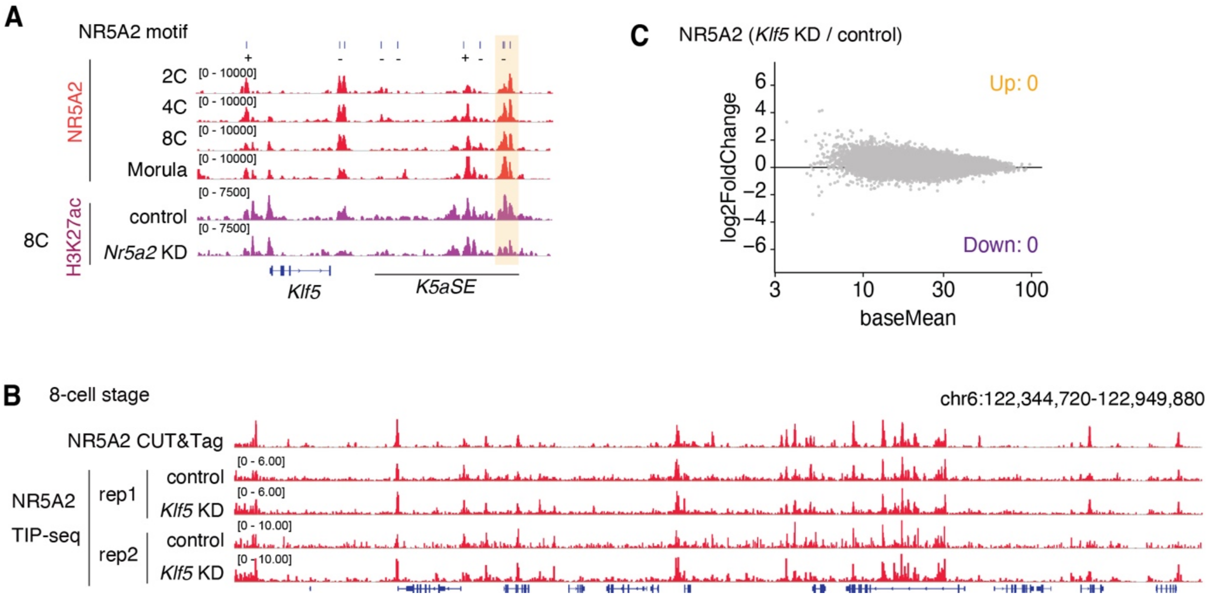

Fig. S6. NR5A2 chromatin binding is largely independent of KLF5.

(**A**) IGV snapshot showing NR5A2 binding near the *Klf5* gene. A highlighted region indicates a decreased H3K27ac on *Klf5*-adjacent distal regulatory elements (K5aSE) (Su et al., 2025). (**B**) IGV snapshot showing NR5A2 CUT&Tag (rep2, 90 8-cell embryos) and NR5A2 TIP-seq (control 8-cell embryos, rep1: 25, rep2: 26. *Klf5* KD 8-cell embryos, rep1: 30, rep2: 30). TIP-seq data are normalized with spike-in DNA. *Klf5* KD efficiency was confirmed by immunofluorescence staining using the residual embryos. (**C**) Differential binding analysis of NR5A2 TIP-seq in *Klf5* KD. The number of increased and decreased NR5A2 binding upon *Klf5* KD is shown.

Development • Supplementary information

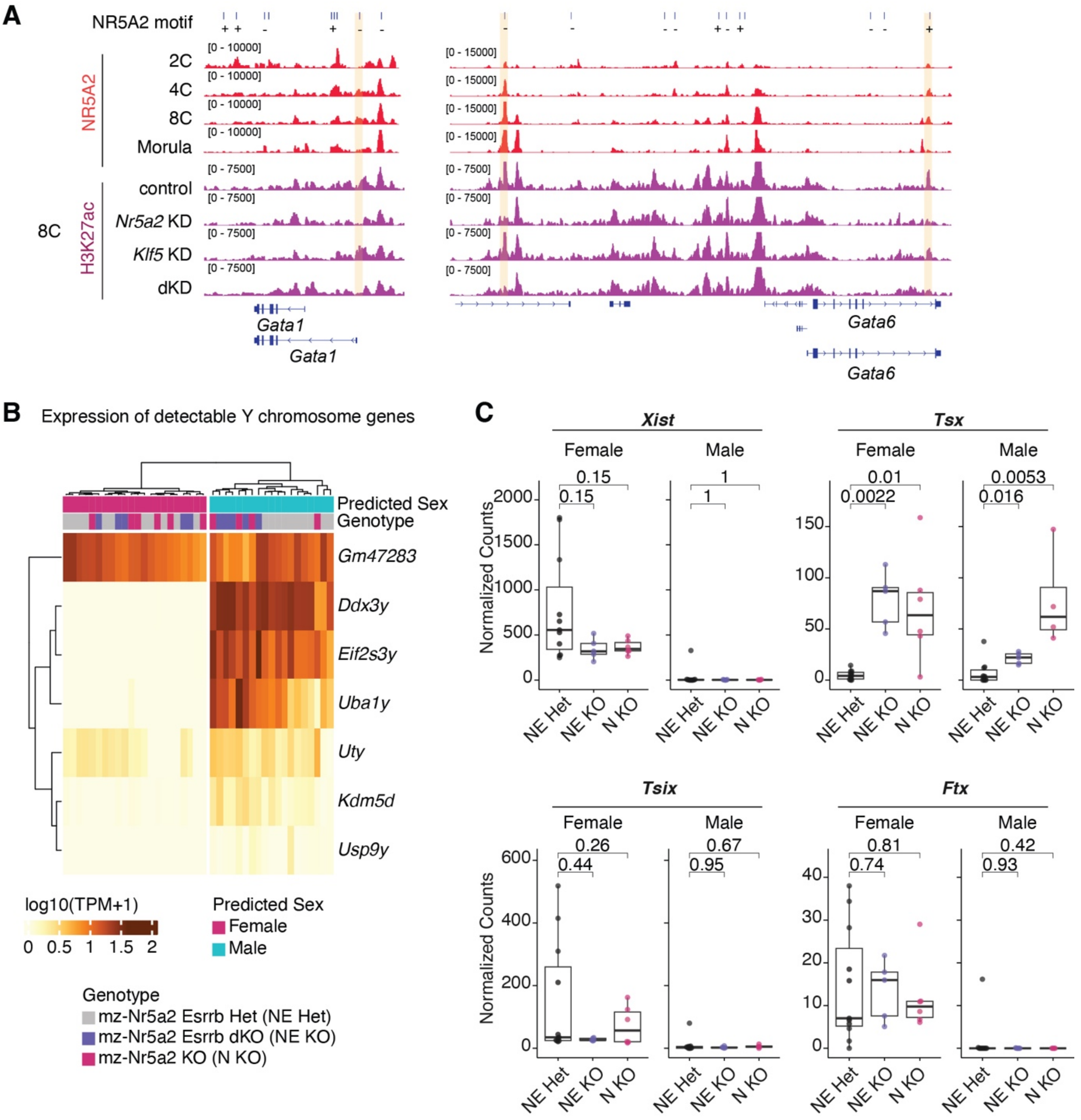

Fig. S7. NR5A2 binding profile near *Gata1* and *Gata6* genes and *Xist*-related gene expression changes from single-embryo RNA-seq data

(**A**) IGV snapshot showing NR5A2 binding near *Gata1* and *Gata6* genes. Highlighted regions indicate decreased H3K27ac signals on NR5A2 binding sites. (**B**) Reanalysis of public single-embryo RNA-seq dataset (Festuccia et al., 2024) with *Nr5a2* maternal-zygotic knockout 8-cell embryos. Prediction of sex type by detectable Y chromosome gene expression. The clusters of red and turquoise correspond to predicted female and male, respectively. (**C**) Box plots showing *Xist* and XCI-related genes in control, maternal-zygotic (mz) *Nr5a2 Esrrb* KO, and mz *Nr5a2* KO 8-cell embryos.

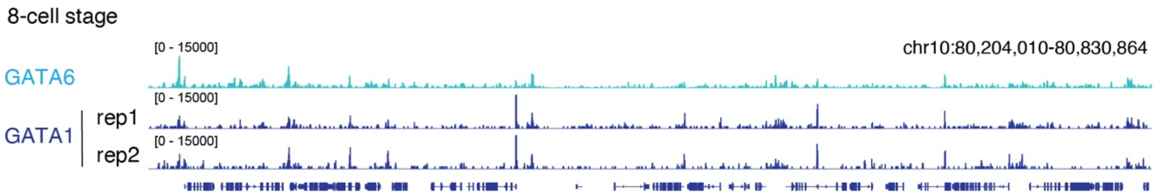

Fig. S8. GATA1 CUT&RUN profile at the 8-cell stage

IGV snapshot showing GATA1 chromatin binding profile (blue) determined by CUT&RUN. The GATA6 CUT&Tag profile (light blue) is shown for comparison.

Development • Supplementary information

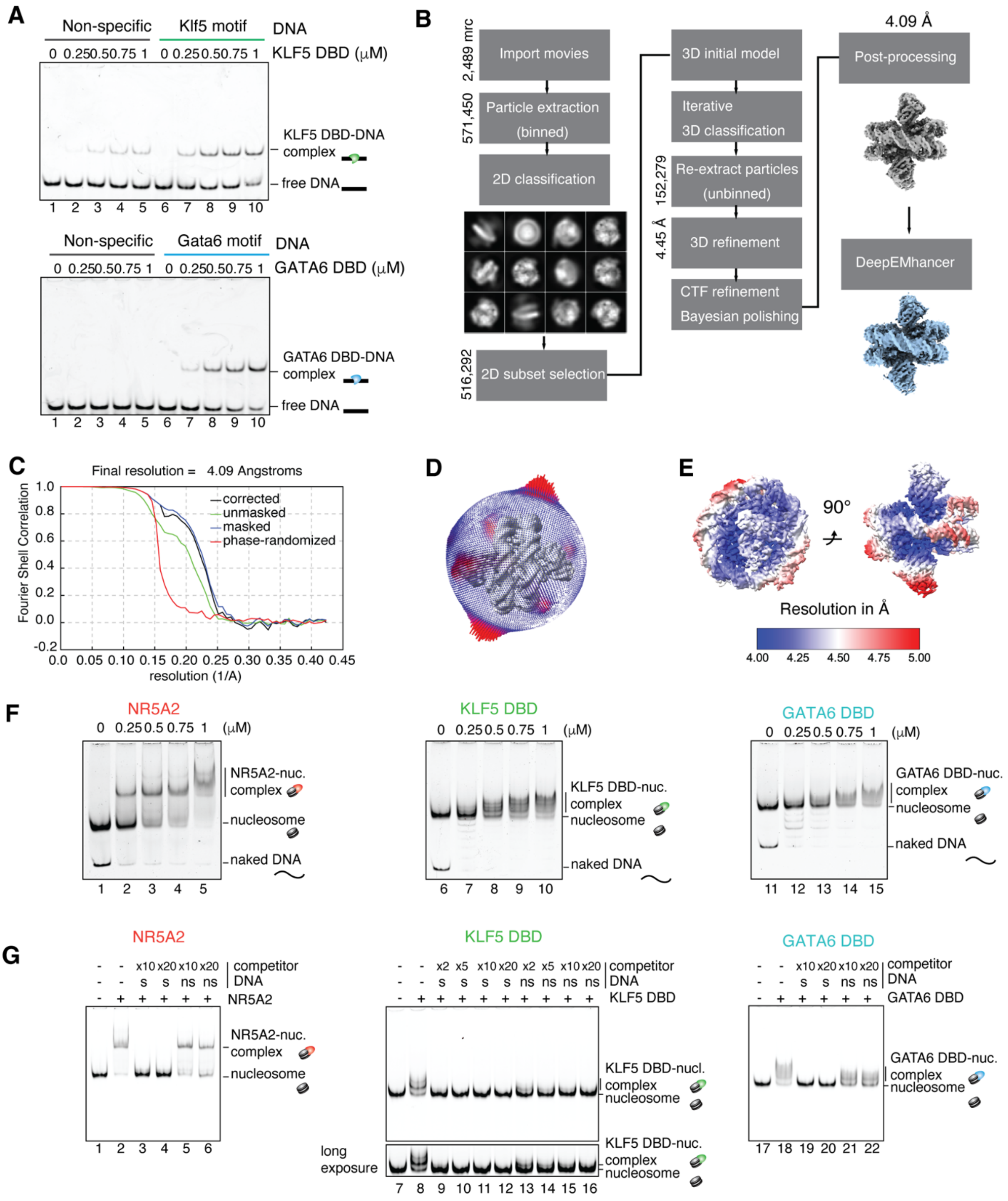

Fig. S9. Cryo-EM data processing and motif specificity of NKG.

(**A**) DNA-binding specificity of KLF5 DBD (left) and GATA6 DBD (right). Three independent experiments were performed, and the reproducibility was confirmed. (**B**) Flowchart of the dataset obtained by Glacios. Cryo-EM map of B1 nucleosome determined by RELION. (**C**) Local resolution of the map of the B1 nucleosome structure. (**D**) Fourier Shell Correlation (FSC) curves of the map. The resolution of the final 3D map was 4.09 Å, as estimated by the gold standard at FSC = 0.143. (**E**) Angular distribution plot of particles employed to reconstruct

maps. (**F**) SYBR gold staining of the gel is shown in Figure 6E. (**G**) Competition assay testing binding specificity. NR5A2, KLF5 DBD, or GATA6 DBD was incubated with B1 nucleosome (50 nM) in the presence of their specific competitor DNA ("s" lanes) or non-specific DNA ("ns" lanes) with indicated amounts. Three independent experiments were performed, and the reproducibility was confirmed.

Table S1. NR5A2 peaks from 2C to morula stages.

Information of NR5A2 peaks in 2C, 4C, 8C, and morula. The mouse genomic loci at NR5A2 peaks are shown.

Available for download at
https://journals.biologists.com/dev/article-lookup/doi/10.1242/dev.205059#supplementary-data

Table S2. ChromHMM input lists.

Public data used for ChromHMM analysis. Accession numbers and cell type are shown.

Available for download at
https://journals.biologists.com/dev/article-lookup/doi/10.1242/dev.205059#supplementary-data

Table S3. The list of differentially expressed genes.

Results of DESeq2 analysis comparing control and knockdown embryos.

Available for download at
https://journals.biologists.com/dev/article-lookup/doi/10.1242/dev.205059#supplementary-data

Development • Supplementary information
